# Self-evaluations and the language of the beholder: objective performance and language solidarity predict L2 and L1 self-evaluations in bilingual adults

**DOI:** 10.1186/s41235-024-00592-4

**Published:** 2024-11-04

**Authors:** Esteban Hernández-Rivera, Alessia Kalogeris, Mehrgol Tiv, Debra Titone

**Affiliations:** 1 Department of Psychology, McGill University, 2001 McGill College Ave., Montreal, Quebec H3A 1G1 Canada; 2 Center for Research Brain Language and Music (CRBLM), Montreal, Quebec Canada; 3 Montreal Bilingualism Initiative (MoBI), Montreal, Quebec Canada; 4 U.S. Census Bureau, Suitland, USA

**Keywords:** Self-evaluative judgements, Bilingual experience, Language attitudes, Language proficiency

## Abstract

People are often asked to self-evaluate their abilities, and these evaluations may not always reflect objective reality. Here, we investigated this issue for bilingual adults’ self-evaluations of language proficiency and usage. We specifically examined how people’s self-reported language solidarity impacted their first- (L1) and second-language (L2) self-evaluations, while statistically controlling for their objective language performance (i.e. LexTALE). We also investigated whether this impact varied for value-laden evaluations (e.g. how “good” am I at my L2) vs. usage-based evaluations (e.g. how often do I use my L2) for two sociolinguistically distinct groups (i.e. English-L1 speakers vs. French-L1 speakers in Montreal). Starting with value-laden self-evaluations, we found that French-L1 speakers with more favourable L2-English solidarity tended to underestimate their objective L2 ability, whereas French-L1 speakers with less favourable L2-English solidarity more accurately estimated their objective L2 ability. In contrast, English-L1 speakers with more favourable L2-French solidarity more accurately estimated their objective L2 ability than those with less favourable L2-French solidarity who underestimated their L2-French abilities. Turning to usage-based self-evaluations, we found that participants' self-evaluations were generally more accurate reflections of their performance, in a manner that was less affected by individual differences in self-reported language solidarity. This implies that language solidarity (or perhaps language attitudes more generally) can implicitly or explicitly impact bilingual adults’ language self-evaluations when these evaluations are value-laden. These data suggest that people’s language attitudes can bias how they perceive their abilities, although self-evaluations based on language use may be less susceptible to bias than those that are value-laden. These data have implications for the study of language and cognition that depend on self-assessments of individual differences and are relevant to work on how people self-assess their abilities generally.

**Supplementary Information:**

The online version contains supplementary material available at 10.1186/s41235-024-00592-4.

## Introduction

We are often asked to evaluate our skills in a manner that is filtered through attitudes about ourselves, others’ abilities, and particular skills in question (e.g. Fleming, 2024; Kruger, 1999; Moore & Small, 2007; Simons, 2013). For example, imagine two people, Veronica and Pauline, who have equally outstanding second-language (L2) skills in French when evaluated objectively. Next, imagine they are both asked to self-evaluate their comparable skills on a scale of 1 to 10 (10 = maximally proficient). Crucially, Veronica has strongly held attitudes about the importance of French and is proud of her hard-won French abilities compared to her friends—she rates herself as an 8. In contrast, while Pauline values French, she does not personally identify with the language to the same degree and has often worried that others judge her French skills as lacking compared to friends—she rates herself as a 6. Thus, despite Veronica and Pauline’s objective equivalence using French, they self-evaluate their skills very differently because unique sociocultural experiences and attitudes lead them to have different standards of evaluation or frames of comparison about their own L2 abilities.

Variability in how we self-evaluate our skills is important to understand because self-evaluation data are often taken as accurate reflections of people’s global skills and experiences (e.g. Alicke et al., 2005; Ehrlinger, 2008; Heilenman, 1990). This is especially pertinent for self-evaluations of language given the close ties between language and a host of socioecological factors (e.g. language attitudes, family and social experience, and cultural identity; see Titone & Tiv, 2023, for a broad overview of socioecological approaches to bilingualism). Relevant here, language attitudes, which are nested within interdependent social spheres of influence (e.g. Feng et al., 2023; Kircher, 2014; Kircher & Zipp, 2022, Titone & Tiv, 2023), may be assessed in general or specific ways (Dragojevic et al., 2021; Giles & Ryan, 1982; Ryan et al., 1982). Prior work suggests that language attitudes are encompassed by and vary along two distinct dimensions—language status and language solidarity (Gardner & Lambert, 1972; Genesee & Holobow, 1989; Ryan et al., 1982). Language status refers to the social prestige of a language within a given sociolinguistic context, whereas *language solidarity* refers to personal identity and sense of belongingness that leads people to favour characteristics of their linguistic in-group (Kircher et al., 2022; Tajfel & Turner, 1986). Thus, language attitudes, self-evaluations, and social identity may be inextricably linked (Giles & Coupland, 1991; Kircher, 2014). Accordingly, people’s solidarity and affinity towards specific languages or language groups within a particular society is reflected by their attitudes about the languages spoken by these groups (e.g. Giles et al., 1973; Giles & Coupland, 1991; Ryan et al., 1982).

In this paper, we extend this approach to investigate how people’s L2 and L1 attitudes about language solidarity and objective proficiency jointly predict their L2 and L1 self-evaluation accuracy. We hypothesize that people’s language solidarity (by virtue of its impact on who we compare ourselves to) can modulate the degree to which objective language performance predicts language self-evaluations (Bosch & Wilbert, 2023; de Bot, 2019). We reason this may occur because one’s language solidarity aligns with cultural norms and expectations of their self-perceived local community (Gullifer et al., 2021; Tomoschuk et al., 2019; Wigdorowitz et al., 2023). Additionally, we address how this varies for different kinds of self-evaluation measures, specifically, those that implicitly require people to compare themselves to others (e.g. proficiency assessments) compared to those that do so to a lesser degree (e.g. frequency of use assessments). Finally, we investigate how the above varies for distinct sociolinguistic groups (i.e. English-L1 and French-L1 bilinguals) who may have different overall patterns of language solidarity within the city of test (i.e. Montreal).

In building to this study, we selectively review what is known about how people self-evaluate their language abilities.

## How do people self-evaluate their own language abilities?

Like many fields, psycholinguistics and bilingualism frequently use individual difference measures of self-reported abilities, usage patterns, or other language-related behaviours (Anderson et al., 2020; Gullifer & Titone, 2020; Kaushanskaya et al., 2020; Veríssimo, 2021). For example, more than 50% of 140 studies published in *Bilingualism: Language and Cognition* relied exclusively on participants’ language proficiency questionnaire data (Hulstijn, 2012). Relevant here, self-reports of language experience rely on people’s self-evaluative judgements, that is, domain-specific perceptions or confidence in their skills, behaviours, or performance (de Bot, 2019; MacIntyre et al., 1997). Self-evaluation measures of proficiency and other behaviours are often gathered through single items on questionnaires that inquire about peoples’ function-specific skills and experiences (de Cat et al., 2023; Kašćelan et al., 2022). For example, many language history instruments have been used to gather self-evaluation data, including the Language History Questionnaire (LHQ; e.g. Li et al., 2020), the Language Experience and Proficiency Questionnaire (LEAP-Q; Kaushanskaya et al., 2020), the Language and Social Background Questionnaire (LSBQ; Anderson et al., 2018), the Bilingual Language Profile Questionnaire (BLP; Birdsong, 2012), and more recently the Contextual Linguistic Profile Questionnaire (Wigdorowitz et al., 2023), among many other questionnaires (*reviewed in*, de Cat et al., 2023).

Interestingly, language self-evaluations, which are a distinct kind of cognitive decision that we make about ourselves, are rarely the dependent variable of interest within bilingualism research. Rather, such measures are typically limited to determining whether a person can be classified as bilingual, and which known language corresponds to a first or second language. Similarly, other studies that emphasize individual differences use self-evaluations as independent variables to predict individual differences in language or other behaviours, often with the goal of comparing across sociolinguistically distinct groups (e.g. Siegelman et al., 2023; Surrain & Luk, 2019; de Bruin, 2017). Crucially, this work assumes that different self-evaluation measures are psychometrically equivalent (i.e. commensurate) to enable comparability across studies—an assumption that may be problematic (for a general review of study commensurability, see Almaatouq et al., 2022; for a commentary specific to bilingualism, see Titone et al., 2024). For example, Nichols, Wild, Stojanoski, Battista, and Owen (2020) classed geographically disparate groups of participants into monolingual and bilingual groups based on a single item from a questionnaire (“*How many languages do you speak?*”), which people may answer differently depending on their internal schema and performance threshold for what constitutes “speaking” a different language for them.

Thus, as previously mentioned, a person’s bilingual language experience may be more or less valid depending on the psychometric and structural validity of a particular measure or latent construct in question (Gullifer & Titone, 2021; Surrain & Luk, 2019; Veríssimo, 2021). Referring to the hypothetical people above, Veronica might more readily and enthusiastically say “yes” to this question than Pauline, not because of her objective language performance, but because Veronica is more likely to personally identify with and have solidarity for her L2 than Pauline. However, very few studies of bilingualism have specifically examined how a person’s attitudes towards particular languages impact their self-evaluations of that language. As noted in Wagner et al. (2022), this issue is particularly problematic when researchers ask participants to quickly classify themselves in broadly defined categories based on their interpretation of questions asking whether they know a language or not, or how skilled they are in that language compared to their own conceptualization of a native-like speaker. Thus, self-evaluation questions can vary in how they are presented or formulated such that some may be more susceptible to subjective bias than others (de Cat et al., 2023; Kašćelan et al., 2022; Rothman et al., 2023), including the language people focus on when making self-directed judgements (e.g. Flake et al., 2022).

In this paper, we reason that self-evaluations vary in at least two broad ways that may be described as either *value-laden* or *usage-based*. Accordingly, some self-evaluations prompt people (implicitly or explicitly) to evaluate their performance for a specific functional dimension (e.g. how proficient are you at speaking/listening/reading/writing in your L2?) or globally (e.g. how proficient are you at generally using your L2?) relative to an implicit gold-standard of maximal language performance (i.e. a “native” speaker). We refer to these self-evaluations as *value-laden* because of the implicit demand for people to compare themselves to others. In contrast, other self-evaluations have lesser social comparison demands in that they people to estimate their *usage-based* experiences, for example, how frequently they use a particular language in specific ways (e.g. what percentage of an average day do you currently use your L2 with friends/family/co-workers/classmates?) or globally (e.g. generally, what percentage of an average day do you currently use your L2?). We refer to these as *usage-based self-evaluations*.

Crucially, the cognitive and psycholinguistic literature often considers both kinds of evaluations as interchangeable or commensurate, an assumption that may not be true (for a general review of study commensurability, see Almaatouq et al., 2022; for a commentary specific to bilingualism, see Titone et al., 2024).

Thus, an open question within bilingualism research, and generally, is whether value-laden self-evaluations are more impacted than usage-based self-evaluation measures by extraneous factors that drive one’s confidence or belief in how their performance compares with others. Addressing this question requires understanding how value-laden and usage-based self-evaluations relate to objective performance measures, and how this relation is modulated by people’s frame-of-reference or attitudes (e.g. Garcia & Gollan, 2022; Gollan et al., 2012). We turn next to this issue, emphasizing a key objective measure frequently used in the literature—the Lexical Test for Advanced Learners of English (LexTALE) (Lemhöfer & Broresma, 2012).

## The LexTALE as a measure of objective language proficiency

The LexTALE was designed to offer a quick, objective assessment of proficiency by assessing people’s receptive lexical knowledge (i.e. vocabulary). Of relevance here, the LexTALE has variably correlated with different measures of L1 and L2 experience (de Bruin et al., 2017; Marian et al., 2007; Lemhöfer & Broresma, 2012), which leaves open the possibility that some people systematically over- or underestimate their language abilities relative to this objective benchmark (for discussion of this point, see Gullifer et al., 2021). For example, Brysbaert (2013) investigated the association between French-L2 and French-L1 bilingual participants’ value-laden (i.e. self-reported proficiency) and usage-based self-evaluations (i.e. years of French instruction at school) with respect to French LexTALE performance. They found that only French speakers who rated themselves as very high or low were successful at accurately assessing their own abilities and concluded that people’s unique frame-of-reference may give rise to different data patterns (see also, Izura et al., 2014).

Similarly, Ferre and Brysbaert (2017) investigated this issue using Spanish LexTALE performance for Spanish-Catalan bilingual adults. They compared value-laden self-evaluations (i.e. self-reported proficiency) and usage-based self-evaluations (i.e. overall usage frequency) for Spanish. An analysis of the entire sample (including both L1 and L2 Spanish participants) revealed a significantly high correlation for both value-laden measures and LexTALE scores, and usage-based measures and LexTALE scores. However, sub-analyses of each L2 and L1 Spanish group alone showed different patterns. For the L2 Spanish group, both types of self-evaluation measures correlated moderately with LexTALE performance. In contrast, for the L1 Spanish group, neither self-evaluation type correlated with LexTALE performance. The authors concluded that the L2/L1 difference in correlation patterns arose because the Spanish L1 group consistently perceived themselves as maximally proficient, in contrast with the more heterogeneous Spanish L2 group who perceived their abilities more in line with their actual performance (see also, Izura et al., 2014).

Collectively, these LexTALE studies suggest that people’s self-evaluations can be systematically impacted by L2 and L1 speakers’ unique frames-of-reference. Moreover, this suggestion is consistent with emerging literature suggesting how sociolinguistic factors (e.g. L2 identity, L2 attitudes) systematically modulate the association between self-evaluative and objective performance-based measures more broadly (de Bot, 2019; Gullifer et al., 2020; Kałamała et al., 2022; Titone & Tiv, 2023; Tomoschuk et al., 2019; Trofimovich et al., 2016; Wagner et al., 2022; Wigdorowitz et al., 2023).

For example, Tomoschuk et al. (2019) tested how two sociolinguistically distinct bilingual groups (i.e. Spanish–English and Chinese-English bilinguals) self-evaluated their dominant language proficiency compared to their objective proficiency using the Multilingual Naming Test (Gollan et al., 2012; i.e. see Analysis 3, pp. 522–525), where dominance was operationalized categorically based on self-rating scores. They found that non-English-dominant bilinguals self-assessed their English ability relatively accurately; however, English-dominant bilinguals overestimated their abilities (results were similar when dominance was assessed continuously). The authors concluded that correlations between self-assessed language proficiency (i.e. value-laden self-evaluations in our view) and objective measures were dependent on people’s unique frame-of-reference (see also, Bonvin et al., 2023).

Similarly, Kałamała, Senderecka and Wodniecka (2022) investigated factors that impact Polish-English bilinguals’ self-reported language experience. In doing so, they assessed value-laden self-evaluations (i.e. self-reported L2 proficiency), usage-based self-evaluations (i.e. daily L2 usage and language entropy), and a composite objective measure of L2 proficiency (i.e. the average score in an oral fluency and vocabulary knowledge tasks). They reported three key findings. First, usage-based but not value-laden L2 self-evaluations related to L2 objective proficiency measures (i.e. a composite vocabulary knowledge score). Second, earlier L2 acquisition led to greater accuracy in self-perceived L2 proficiency, and later L2 acquisition led to reduced objective accuracy. Finally, increased language entropy (i.e. a usage-based measure of how people distribute the use of their languages in daily life) patterned with higher self-reported L2 ability (see also, Gullifer & Titone, 2020). Kałamała et al. concluded that bilinguals’ understanding of self-assessed language proficiency (i.e. the very meaning behind the concept of language proficiency) depends on participants’ early language history and sociolinguistic patterns of usage (see also, Gullifer et al., 2021).

## The present study

The work reviewed above suggests that the capacity to self-evaluate our language abilities depends partially on our unique frame-of-reference, which are themselves impacted by a wide array of contextual, attitudinal, and individual differences (e.g. Titone & Tiv, 2023). Thus, we hypothesized that people’s language solidarity (by virtue of its impact on whom we compare ourselves to) can modulate the degree to which objective language performance predicts language self-evaluations (Bosch & Wilbert, 2023; de Bot, 2019). We reason this may occur because one’s language solidarity aligns with cultural norms and expectations of their self-perceived local community (Gullifer et al., 2021; Tomoschuk et al., 2019; Wigdorowitz et al., 2023). We investigate this issue explicitly by examining how individual differences in people’s language solidarity towards their L2 and L1 (which directly relate to their frame-of-reference) predicted L2 and L1 value-laden vs. usage-based self-evaluations, over and above the expected impact of participants’ L2 and L1 objective language performance (i.e. LexTALE). We also examined whether these differences varied across different sociolinguistic groups, specifically, English-L1 vs. French-L1 bilinguals residing in Montreal.

## Methods

### Participants

We tested 62 French–English bilinguals who self-identified as fluent in both French and English and regularly used both languages, which is a subsample of Feng et al. (2023) who had complete data for this purpose. Participants reported living in Canada since birth (n = 41) or within the province of Quebec (n = 31). Of the participants, not born within Canada, the average amount of time spent living within the francophone province was 5.45 years. Beyond English and French, some participants also reported additional knowledge of other languages, with the most common language being Spanish (see Tables S2 and S3 in supplemental materials).

Crucially, participants differed in when they acquired their L1 and L2—18 people acquired the L2 at or before 1 year of age (i.e. simultaneous bilinguals), and 44 people acquired their L2 at later stages in life (i.e. sequential bilinguals who were either English- or French-dominant). Of the simultaneous bilinguals, ten tended towards French dominance, and eight tended towards English dominance, which was determined based on their language of instruction history and their self-declared language preference (i.e. the language in which they felt most comfortable). Thus, we divided our overall sample of 62 participants into subgroups of 38 French-L1 and 24 English-L1 bilingual adults. See Table 1 for participant summary information.

**Table 1:** Descriptive statistics of participant sample and computed self-evaluation measures

Variable	French-L1 Bilingual Adults (n = 38)			English-L1 Bilingual Adults (n = 24)		
	Mean	SD	Range	Mean	SD	Range
L2 AoA (years)	4.92	3.96	0.00–12.00	3.46	3.15	0.00–12.00
IV #1: Objective Language Ability						
*L2 LexTALE (% accuracy)*	82.88	9.68	57.5—98.70	62.61	10.07	44.60—86.60
*L1 LexTALE (% accuracy)*	78.25	10.03	53.5—95.50	86.98	8.29	68.70—98.70
IV #2: Attitudinal frame-of-reference						
*L2 Attitudinal language solidarity*	4.70	1.10	2.67–7.00	4.29	1.18	1.30—6.50
*L1 Attitudinal language solidarity*	5.13	1.01	2.78–7.00	5.23	0.70	3.80—6.70
DV #1: Value-laden self-evaluations (self-reported proficiency)						
*L2 Value-laden self-evaluations*	− 0.26	1.07	− 2.60—0.93	− 0.54	1.05	− 2.10—0.90
*L1 Value-laden self-evaluations*	0.40	0.76	− 2.30—0.90	0.46	0.68	− 2.10—0.90
DV #2: Usage-based self-evaluations						
*L2 Overall language use*	0.54	0.23	0.00—0.90	0.21	0.19	0.00—0.80
*L1 Overall language use*	0.44	0.23	0.00—0.90	0.79	0.19	0.00—0.90

The scale ranges for the reported measures are as follows: (a) Attitudinal language solidarity: 1–7, (b) Value-laden self-evaluations: -3–3, (c) Usage-based self-evaluations: 0–1 values reflecting usage proportions

## Procedure and materials

Participants completed questionnaires and tasks to assess their language experience and abilities. First, participants completed a French (Brysbaert, 2013) and English (Lemhöfer & Broersma, 2012) version of the LexTALE task, programmed on OpenSesame (i.e. the objective language assessment task; Mathôt et al., 2012). Then, participants completed a language attitudes questionnaire (Feng et al., 2023), from which we derived a language solidarity index as our frame-of-reference measure. Finally, participants completed a Language and Social History Questionnaire, custom to our laboratory, which included L2 and L1 value-laden and usage-based self-evaluation questions. Participants received compensation of $10 CAD per study-hour or equivalent course credit, as approved by the McGill Research Ethics Board.

Based on these tasks and questionnaires, we first identified two baseline language measures to serve as independent variables across models: L2 and L1 LexTALE accuracy, and L2 and L1 language solidarity frame-of-reference index. Then, we extracted L2 and L1 value-laden and usage-based self-evaluation measures, which served as dependent variables across models. The details of how these variables were designated are presented below.

### Independent variable type #1: L2 and L1 objective language ability (i.e. LexTALE)

We used computerized versions of the English LexTALE designed by Lemhöfer and Broersma (2012), and the French LexTALE designed by Brysbaert (2013), implemented in OpenSesame v3.3.14. LexTALE has high reliability and internal/external validity among various bilingual samples and languages (e.g. English, French, Italian, Spanish, Portuguese, Dutch, Basque; Brysbaert, 2013; Ferre & Brysbaert, 2017; Izura et al., 2014; Lemhöfer & Broersma, 2012). Participants first performed practice trials to ensure task familiarity. For the French LexTALE, participants saw 56 French words and 28 French-looking non-words*.* For each trial, participants were asked to decide, using a keyboard, whether each stimulus was a real French word or not. For the English LexTALE, participants followed a similar procedure, in that they saw 60 words (40 real English words, 20 English-looking non-words), each presented individually. We computed participants’ English and French LexTALE accuracy using formula (1).

1 $$\text{LexTALE }\left({\%}\right)\,=\,\frac{\left(\frac{{\text{N}}{\text{words}}}{\text{TOT words}}{*100}\right) + \left(\frac{\text{Nnonwords}}{{\text{TOTnonwords}}}{*100}\right)}{2}$$

This formula corrects for the unequal proportion of words to non-words across the two language versions and is widely used and easily interpreted (Brysbaert, 2013; Izura et al., 2014; Lemhöfer & Broersma, 2012; Siegelman et al., 2023).

### Independent variable type #2: L2 and L1 solidarity

Participants completed a language attitudes questionnaire, described in Feng et al. (2023), which measured language-specific attitudes towards the L2 and L1. This questionnaire had 48 total questions that broadly probed language-specific attitudes towards English and French (e.g. *I feel a strong sense of community when using French*) and about language laws in Québec, Indigenous and minority languages, and bilingualism generally. In contrast with Feng et al. (2023), which considered responses on the entire questionnaire, we selected a subset of L1- and L2-specific questions that comparably assessed language solidarity across English and French (e.g. *French gives me a strong sense of community*). This allowed us to derive two continuous indices of participants’ attitudinal language solidarity towards the L2 and L1, which we took as a proxy for their self-evaluation frame-of-reference. To compute these indices, we calculated the mean score using participants’ responses to all subset questions (see Table 2, see Table S1 in supplemental materials for the complete set of questions).

**Table 2:** Example subset of questions indexing attitudinal language solidarity

Question
Knowing *Lx* is an important part of my personal identity
Speaking *Lx* gives me a sense of community
I like who I am when I speak *Lx*
Speaking in *Lx* increases the value and prestige of what I say
*Lx* makes me feel secure
I feel true to myself when I speak *Lx*

Lx is used as a placeholder. In the questionnaire, participants saw either English or French in place of Lx

### Dependent variable type #1: L2 and L1 value-laden self-evaluations

Participants completed an exhaustive language background questionnaire customized for our laboratory that probed bilingual experience. For the value-laden assessment, participants self-evaluated their L2 and L1 abilities across modalities, using the prompt: *“Please rate your current ability in [reading, writing, speaking, and listening] for [all languages that you know]*”. To respond, participants used the following scale (i.e. *1—Non-existent; 2—Poor; 3—Limited; 4—Functional; 5—Good; 6—Very Good; 7—Maximal Proficiency*).

**Fig. 1:**
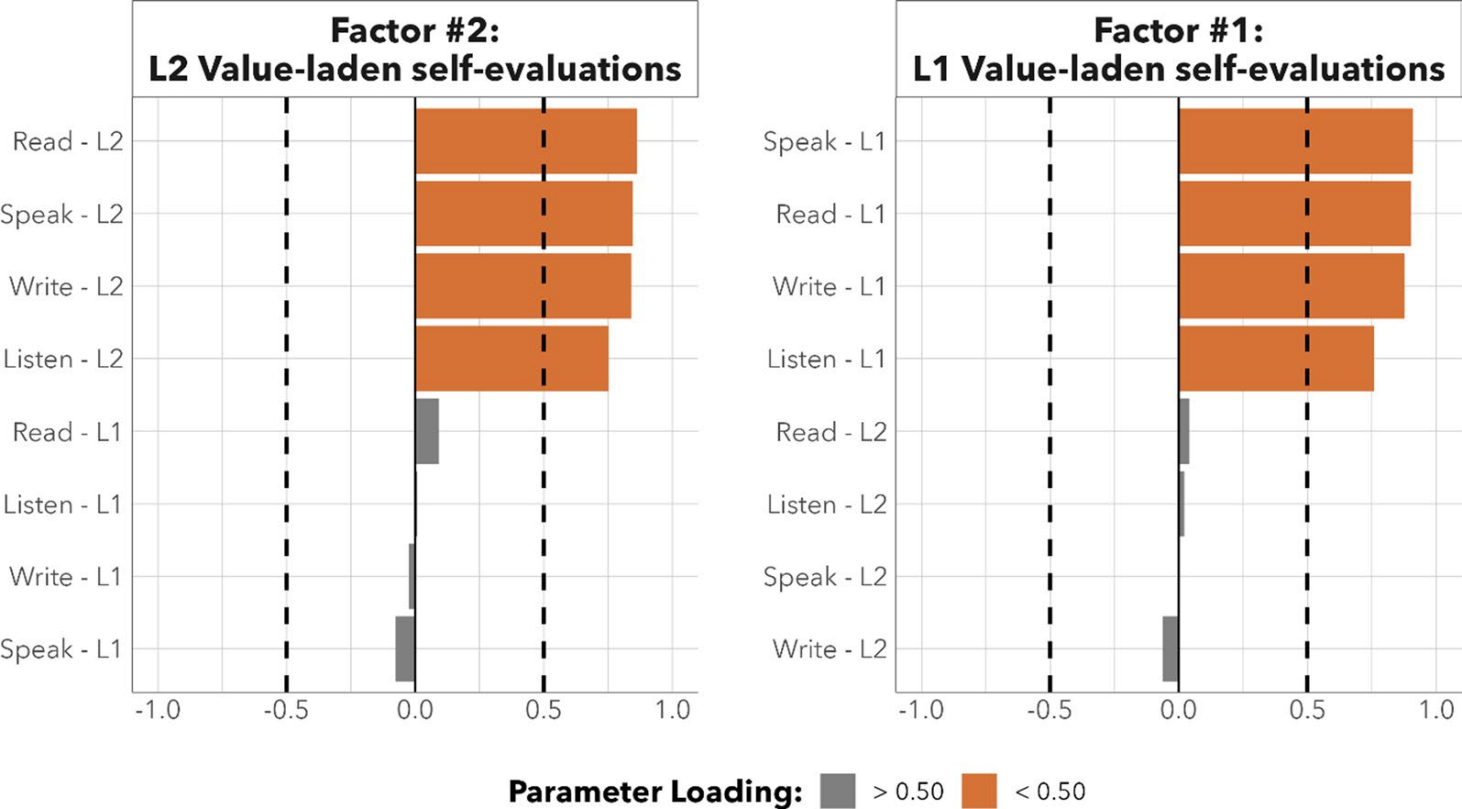
Factor loadings that reflect L1 and L2 value-laden self-evaluations

L2 and L1 value-laden self-evaluations were thus assessed as two continuous variables representing participants’ general language abilities. We then conducted an exploratory factor analysis (EFA) on these data to assess general self-evaluation patterns across modalities. To determine the appropriate number of factors to extract from the best-fitting model, we utilized the parameters, psych, and GPArotation R packages. Goodness of fit metrics indicated that the correlation matrix was factorable, and no items dropped below the 0.50 threshold for sampling accuracy (Bartlett’s *x*^2^(46) = 345.23, *p* < 0.001; KMO = 34.578). We used an oblimin rotation and a maximum likelihood factoring method. This analysis determined that a two-factor model was ideal for extracting separate scores that reflected participants’ L2 and L1 value-laden self-evaluations of proficiency. The resulting two-factor structure accounted for 63.4% of the total variance in the original data. Factors were correlated at |*r*|= 0.40. As illustrated in Fig. 1, the first factor (L2 value-laden self-evaluations: 30.54% of total variance) reflected participants’ L2 self-perception and self-confidence. Meanwhile, the second factor (L1 value-laden self-evaluations: 31.20% of total variance) reflected participants’ L1 self-perception and self-confidence. Thus, from the EFA, we extracted two separate factor scores for participants’ L2 and L1, which we used as dependent variables in subsequent models. Higher values in these factor scores indicate that participants self-evaluated their overall L2 or L1 abilities in a more favourable manner.

### Dependent variable type #2: L2 and L1 usage-based self-evaluations

For the usage-based assessment, participants self-evaluated the percentage of time they perceived using each of their known languages in general when thinking about their usage across different social contexts. Thus, for each participant, their self-estimated general language usage had a lower limit of 0%, indicating that the participant self-perceived *“not using a language at all,”* and an upper limit of 100%, indicating that the participant estimated that the language was “*used all the time*”. For our analyses, we converted participants’ L2 and L1 percentage-based responses to proportions and used them as separate dependent variables.

## Results

All models and figures were generated in R (R Core Team, 2022). To conduct these analyses, we used the following packages: *robustbase* to generate the fitted models (Version 1.1.27.1), *parameters* to evaluate the significance of the model estimates using a *Wald* approximation (Version 3.1.3), *effects* for model predictions (Version 4.2.0), and *ggplot2* for data visualization (Version 2.3.5). The data and scripts may be found on the Open Science Framework at: https://osf.io/bzra8/.

### How do language solidarity and objective performance predict L2 self-evaluations?

To address this question, we constructed separate robust linear regression models for participants’ value-laden and usage-based self-evaluations of their L2. For both models, we included fixed effects for their objective L2 language performance (i.e. LexTALE scores, scale-centred at 0), L2 language solidarity (scale-centred at 0), language group (French-L2/English-L1 vs. English-L2/French-L1, effects coded at -0.5/0.5), and their interactions. The first model was specified with L2 value-laden self-evaluations as the dependent variable (see left side of Table 2), whereas a second model was specified with L2 usage-based self-evaluations as the dependent variable (see right side of Table 2).

Inspection of the L2 value-laden self-evaluations model (see Table 3) showed a main effect of L2 objective performance but not for language group nor their interaction, indicating that participants’ L2 self-evaluations were predicted to some degree by their objective L2 scores across both groups of participants (β = 0.44, SE = 0.17, t(54) = 2.62, *p* = 0.011). Furthermore, in line with our hypotheses, we found evidence for a large positive effect of L2 solidarity, indicating that greater L2 solidarity was associated with more positive L2 self-evaluations (β = 0.71, SE = 0.14, t(54) = 4.90, *p* < 0.001). Importantly, the three-way interaction of L2 LexTALE, L2 solidarity, and group was also significant, indicating that people’s L2 value-laden self-evaluations were modulated by the joint impact of participants’ L2 objective performance and L2 language solidarity, with this effect varying slightly across language groups (β = − 0.57, SE = 0.28, t(54) = − 2.09, *p* = 0.042).

**Table 3:** Model comparison tables for L2 value-laden and L2 usage-based self-evaluations

	L2 Value-laden Self-evaluations				L2 Usage-based Self-evaluations			
Model Parameters	β	SE	t(54)	*p*	β	SE	t(54)	*p*
(Intercept)	− 0.24	0.16	− 1.54	0.129	**0.4**	**0.04**	**9.65**	** < 0.001**
LexTALE	**0.44**	**0.17**	**2.62**	**0.011**	**0.16**	**0.04**	**4.36**	** < 0.001**
Lang. Solidarity	**0.71**	**0.14**	**4.90**	** < 0.0001**	**0.09**	**0.03**	**2.94**	**0.005**
Lang Grp	− 0.46	0.31	− 1.46	0.149	0.07	0.08	0.88	0.382
LexTALE * Lang. Solidarity	0.09	0.14	0.64	0.523	**0.08**	**0.03**	**2.81**	**0.007**
LexTALE * Lang. Grp	− 0.14	0.31	− 0.45	0.653	− 0.01	0.07	− 0.15	0.885
Lang. Solidarity * Lang. Grp	− 0.49	0.29	− 1.71	0.094	− 0.10	0.06	− 1.82	0.074
LexTALE * Lang. Solidarity * Lang. Grp	− **0.57**	**0.28**	− **2.09**	**0.042**	0.02	0.05	0.39	0.698

Significant model coefficients are depicted in bold

**Fig. 2:**
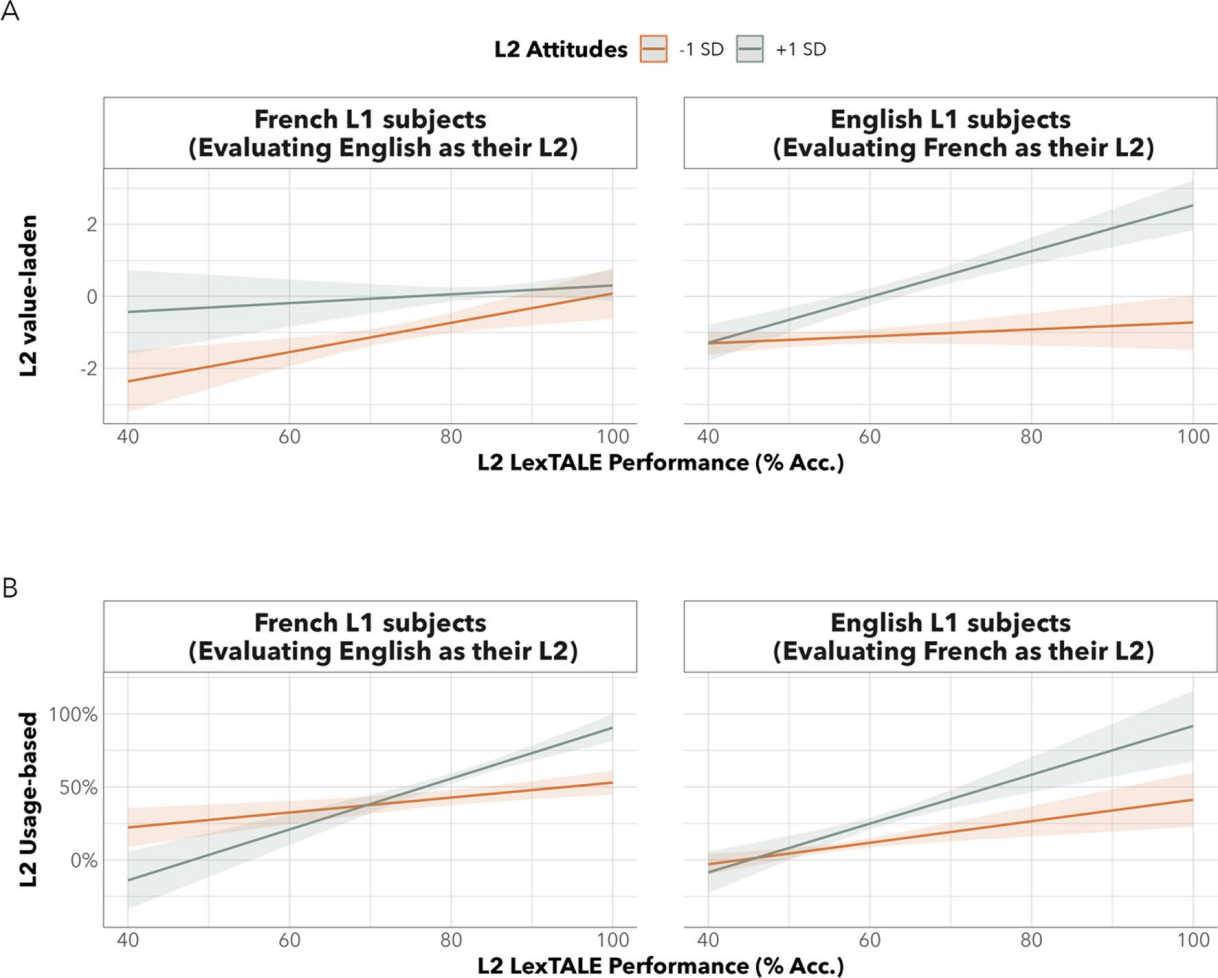
Partial effects plots for the impact of language attitudes on L2 value-laden and usage-based self-evaluations. Across all panels, thick lines represent the model-based predictions, with shaded areas representing + / − 1 SEM

To further examine this interaction, we evaluated each language group separately (English-L1 participants evaluating French as their L2, and French-L1 participants evaluating English as their L2). Interestingly, the interaction between L2 solidarity and L2 LexTALE performance was significant for the former, but not the latter group. As shown in Fig. 2 (panel A, right side), this seemed to suggest that English-L1 participants with greater L2 solidarity towards French had a tighter coupling between their objective L2 performance and their L2 self-evaluations compared to participants with lower L2 solidarity. Meanwhile, for French-L1 participants evaluating English as their L2, we found only a main effect of L2 solidarity, but no interaction between L2 solidarity and L2 performance (see Panel A, left side).

For L2 usage-based self-evaluations, we also probed the effect of L2 LexTALE, L2 solidarity, and language group. Inspection of the model (Table 2, right side) showed evidence of a main effect of L2 LexTALE, indicating that participants’ perceived L2 usage was strongly associated with their objective L2 LexTALE performance scores (β = 0.16, SE = 0.04, t(54) = 4.36, *p* < 0.001). Interestingly, we also found evidence for a small effect of L2 language solidarity, indicating that greater solidarity towards the L2 was also associated with the overall proportion of time participants perceived using their L2 on an average day (β = 0.09, SE = 0.03, t(54) = 2.94, *p* = 0.005). Here too, we found no main effect of language group (β = 0.07, SE = 0.08, t(54) = 0.88, *p* = 0.382).

Additionally, we found a significant two-way interaction between L2 solidarity and L2 LexTALE, indicating that participants’ solidarity towards their L2 impacted the coupling between their L2 usage-based evaluations and objective L2 ability scores (β = 0.08, SE = 0.03, t(54) = 2.81, *p* = 0.007). As depicted in panel B of Fig. 2, the coupling between L2 LexTALE scores and L2 usage-based evaluations decreased as participants’ solidarity towards the L2 trended negative. Importantly, the three-way interaction was not significant, indicating that the impact of L2 language solidarity on the coupling between L2 usage-based self-evaluations and L2 performance was comparable for the two sociolinguistically distinct groups of bilingual participants (i.e. English-L1 vs. French-L1).

### How do language solidarity and objective performance predict L1 self-evaluations?

To answer this question, we constructed two additional robust linear regression models. For both models, we included fixed effects for their objective L1 objective performance (i.e. LexTALE scores, scale-centred at 0), L1 solidarity (scale-centred at 0), language group (French-L2/English-L1 vs. English-L2/French-L1, effects coded at -0.5/0.5), and their interactions. The first model was specified with L1 value-laden self-evaluations as the dependent variable (see left side of Table 3), whereas the second model was specified with L1 usage-based self-evaluations as the dependent variable (see right side of Table 4).

**Table 4:** Model comparison tables for L1 value-laden and L1 usage-based self-evaluations

	L1 Value-laden Self-evaluations				L1 Usage-based Self-evaluations			
Model Parameters	β	SE	t(54)	*p*	β	SE	t(54)	*p*
(Intercept)	**0.73**	**0.04**	**17.4**	** < 0.001**	**0.65**	**0.03**	**24.23**	** < 0.001**
LexTALE	− 0.01	0.07	− 0.10	0.919	**0.07**	**0.02**	**3.31**	**0.002**
Lang. Solidarity	− 0.02	0.05	− 0.44	0.660	0.07	0.04	1.77	0.083
Lang Grp	0.05	0.08	0.68	0.502	− **0.34**	**0.07**	− **5.21**	** < 0.001**
LexTALE * Lang. Solidarity	− 0.02	0.10	− 0.17	0.863	− 0.03	0.02	− 1.26	0.214
LexTALE * Lang. Grp	0.24	0.15	1.55	0.126	**0.14**	**0.04**	**3.07**	**0.003**
Lang. Solidarity * Lang. Grp	0.01	0.10	0.08	0.938	− 0.02	0.07	− 0.24	0.809
LexTALE * Lang. Solidarity * Lang. Grp	− 0.34	0.19	− 1.78	0.081	0.07	0.06	1.16	0.250

Significant model coefficients are depicted in bold font

**Fig. 3:**
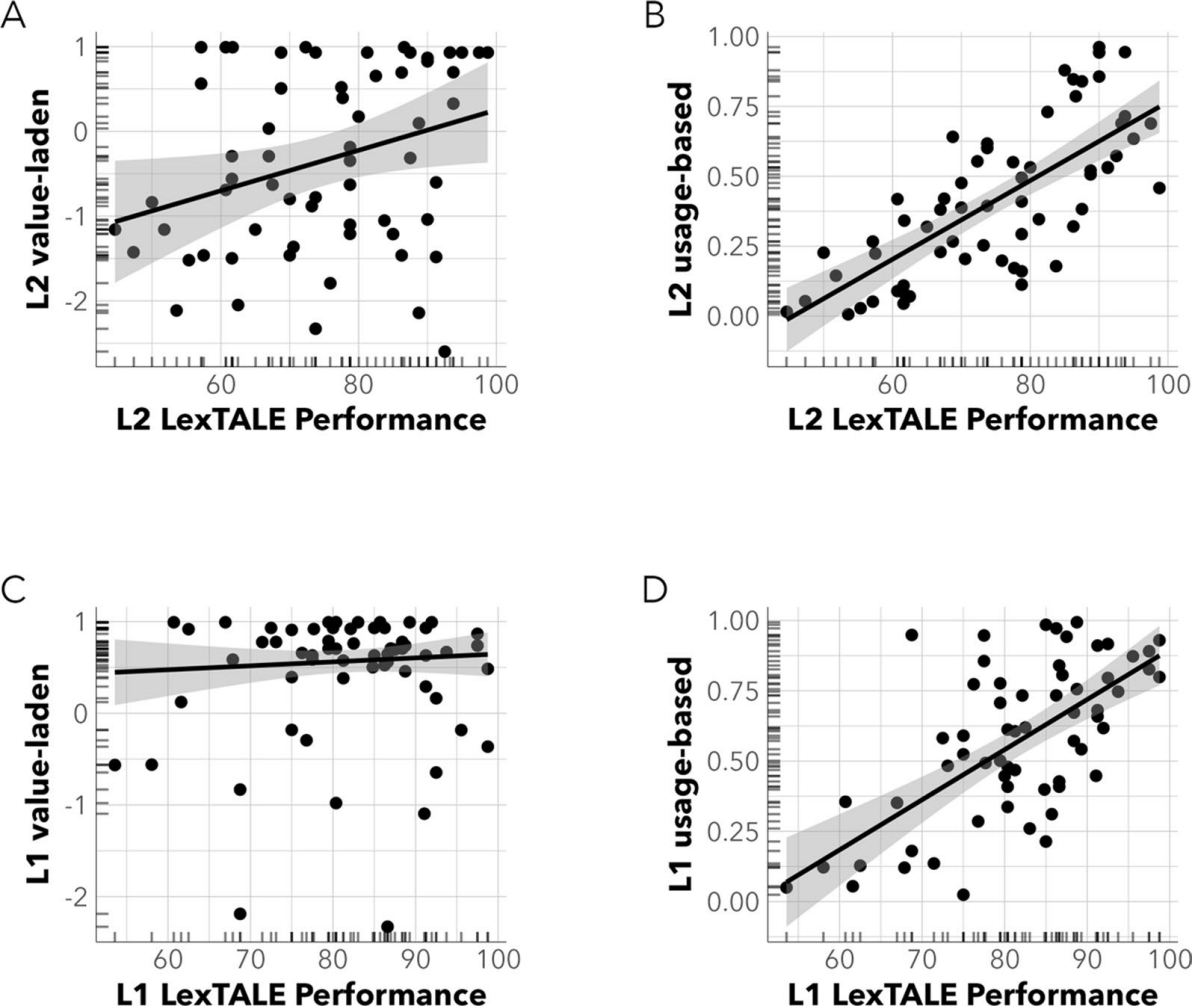
Scatterplots of pairwise associations between value-laden and usage-based measures and LexTALE performance scores

First, we examined the effect of L1 LexTALE performance, L1 solidarity, and language group on value-laden self-evaluations of L1 ability. Inspection of the model showed there was no main effect of L1 objective performance on value-laden self-evaluations of L1 ability (β = − 0.01, SE = 0.07, t(54) = − 0.10, *p* = 0.919). Similarly, there was no effect of L1 solidarity (β = − 0.02, SE = 0.05, t(54) = − 0.44, *p* = 0.660) nor language group (β = − 0.05, SE = 0.08, t(54) = − 0.68, *p* = 0.502) on self-evaluations of L1 ability (see right side of Table 3). We also found no evidence for any of the interaction effects of interest. Crucially, further inspection of the data revealed that participants consistently self-reported a degree of maximal proficiency, regardless of whether their objective L1 LexTALE performance was high or low (see Fig. 3, panel C). We discuss the relevance of this finding in the conclusions section (see also Table 5).

**Table 5:** Abridged summary of results

	L2 Self-evaluations		L1 Self-evaluations	
	Value-laden	Usage-based	Value-laden	Usage-based
Impact of LexTALE performance	✓	✓	X	✓
Impact of language solidarity	✓	✓	X	X
Impact of Language Group	✓	X	X	✓
Joint impact of language performance & solidarity	✓	X	X	X

Summary of results across the four reported regression models. A checkmark (✓) indicates an overall significant main or interaction effect for a particular variable, whereas a cross (X) indicates a non-significant effect at the alpha level of 0.05. Tables 3 and 4 report the non-abridged summary of the results shown here

Finally, we examined the effect of L1 LexTALE, L1 language solidarity, and language group on L1 usage-based self-evaluations. Here, we found a robust effect of L1 objective performance (β = − 0.07, SE = 0.02, t(54) = − 3.31, *p* = 0.002), indicating that participants’ self-evaluations of general L1 usage were strongly associated with their L1 objective performance scores. Moreover, we found no significant effect of L1 solidarity (β = − 0.07, SE = 0.04, t(54) = − 1.77, *p* = 0.083), indicating that the aforementioned relationship between participants’ self-perceived L1 usage patterns and their objective L1 performance scores in the LexTALE task remained largely unaffected by participants’ degree of attitudinal solidarity towards their L1.

## Discussion

We examined several factors that impact people’s language self-evaluations across the L2 and the L1. We were specifically interested in determining how people’s language-relevant frames-of-reference, indexed by the L2 language attitude of solidarity, modulated the accuracy of their L2 self-evaluations over and above what would be expected given their objective L2 ability. We were also interested in determining whether L2 value-laden self-evaluations that implicitly require people to compare themselves to others were more susceptible to bias than L2 usage-based self-evaluations that may do so to a lesser degree. Lastly, we examined whether the overall pattern of findings for L2 self-evaluations extended to people’s more practised L1.

The ***first key finding*** was that the association between people’s objective L2 performance and value-laden L2 self-evaluations was modulated by L2 solidarity in a manner that varied for English-L1 and French-L1 participants. In the case of French-L1 participants, those who reported greater solidarity towards their L2 (English) underestimated their objective L2 ability, whereas those who reported lesser solidarity towards their L2 more accurately estimated their objective L2 ability. In contrast, English-L1 participants who reported greater solidarity towards their L2 (French) showed a tighter coupling between their objective and self-perceived L2 ability, whereas participants who reported less solidarity towards their L2 showed a decoupling between their self-perceived and objective L2 ability. For instance, English-L1 bilinguals who held lower solidarity towards French were more likely to underestimate their objective abilities compared to their L2 performance. Whether this effect replicates for larger and more heterogeneous samples is a question that we are currently pursuing.

Collectively, these data suggest that people’s feelings of language solidarity may have impacted their general attitudinal frame-of-reference about who they compare themselves to (or who they are being judged by), which in turn impacted the accuracy of their L2 value-laden self-evaluations. This is consistent with suggestions from past work that people’s language attitudes should lead them to up- or down-weight their language self-evaluations, over and above their objective abilities, in a manner that aligns with the cultural norms and expectations of the local community (Gullifer et al., 2021; Tomoschuk et al., 2019; Wigdorowitz et al., 2022). Accordingly, individual differences in language attitudes systematically shape how people perceive their abilities because a person’s perception and solidarity towards the in-group members of that language group change who they compare themselves to. Consequently, the more positively a person perceives their L2, the more favourable they would evaluate their abilities, potentially leading to a misrepresentation of their objective language ability. Consistent with this idea, we found that self-evaluated L2 ability was not as strongly associated with L2 objective performance once we considered the moderating role of participants’ L2 solidarity.

The results for value-laden self-evaluations also extend prior work on bilingualism concerning the imprecision of participants’ self-reports of their language abilities, as well as the potential imprecision of existing proxy measures commonly used to measure bilinguals’ language proficiency (e.g. Tomoschuk et al., 2019). For example, our findings resemble those reported by Gullifer et al. (2021) for a similar sample of French–English bilinguals. Specifically, Gullifer and colleagues observed that participants’ perceptions about their language ability differed considerably from their objective performance when they were asked to self-assess their L1. Here, we not only found evidence replicating this effect for participants’ L1 abilities, but also found evidence of a similar effect with respect to participants’ L2 abilities.

To date, many studies have examined the accuracy and consistency of self-reported proficiency measures in relation to various objective language proficiency measures such as LexTALE, semantic fluency, verbal fluency, and picture naming tasks. However, a significant portion of these studies (c.f., Garcia & Gollan, 2022; Gullifer & Titone, 2021; Tomoschuk et al., 2019) have not fully considered the idea that self-reported proficiency measures may not necessarily provide unbiased estimates of one’s language abilities (see Fig. 3, panels A and C).

Rather, they may reflect an individual’s confidence in their language skills relative to those of their in-group peers. Our findings support the idea that self-evaluative judgements of language ability can be systematically swayed by people’s beliefs about how their abilities compare to the abilities of people whom they consider to be their in-group peers. Indeed, as Brysbaert (2013) observed, the lack of reliability of language-specific self-evaluations of proficiency can often be attributed to the idea that many participants’ frame-of-reference when self-reporting their language proficiency hinges on a rather narrow group of comparison. Here, we provide empirical evidence that self-perceptions of language ability may depend on the attitudinal profile of the bilingual population being sampled, as some participants may tend to rate themselves higher or lower to align with cultural expectations and norms (Tiv et al., 2022).

The ***second key finding*** of this study concerns participants’ L2 usage-based self-evaluative judgements. Similar to what was found for value-laden self-evaluation, we again found a strong positive correlation between participants L2 LexTALE performance and their usage-based self-evaluations of overall L2 usage (see Fig. 3, panel B), even after accounting for a small modulating effect of participants’ attitudes towards their L2 (see Fig. 2, panel B). Altogether, this indicated that the strong positive correlation between participants’ objective L2 performance and their L2 usage-based evaluations decreased slightly when attitudes towards the L2 were less favourable, and that this effect was similar for the group of English-L1 and French-L1 participants.

These results align with our expectations that when participants are asked to make usage-based self-evaluations of their contextual language use patterns, there is no frame-of-reference to which they must compare themselves that can strongly bias their mental estimations of their self-reported language usage behaviours. The results show, in line with prior LexTALE validation studies, that participants’ usage-based self-evaluations and their performance-based scores are strongly correlated, and that this pattern remains relatively unaffected by individual differences in bilinguals’ language solidarity even when comparing two sociolinguistically distinct groups of French-native and English-native Montréalers. Notably, both of these effects closely resemble previously reported findings on the impact of individual differences in bilinguals’ language background (Gullifer et al., 2021). For example, Kalamala et al. (2022) found that an objective, composite index of vocabulary knowledge was related to self-reported daily usage of the L2, but that the sooner people acquired their second language, the more their confidence in using an L2 translated into actual knowledge of the vocabulary of that language. Our results further extend these findings by showing that socially grounded aspects of a person’s language background are important when establishing the association between people’s self-evaluations and their objective language abilities.

Furthermore, our results align with Wagner et al. (2022), who addressed the idea that not only language-related self-evaluations, but also evaluations of others’ abilities can be impacted by societal judgement biases, that is, people’s perceptions of what constitutes a language and a bilingual. Specifically, Wagner and colleagues tested whether descriptions (e.g. presence or absence of a writing system; relatedness to another known language; and geographic specificity) of a hypothetical person’s linguistic competence and language background were interpreted by participants as qualifying as bilingual (e.g. To address the question “who is bilingual,” participants rated 20 fictional language use and proficiency scenarios on a scale from “Monolingual” (0) to “Bilingual” (10).). Crucially, the authors found that fictional individuals were judged to be more bilingual when they learned their L2 earlier in life, when they engaged in continued rather than sparse usage of their L2, as well as when both the L2 and L1 languages of a person had a written system. Taken together, these data offer evidence of a large degree of variability for what constitutes a language or what criteria participants rely on when attempting to judge whether they or someone else is bilingual or not. The authors concluded that their results were consistent with the notion that language-specific differences in people’s experiences are key factors to consider when asking people to evaluate not only themselves but also the profile of others (Gullifer et al., 2021; Tiv et al., 2022).

The ***final key finding*** of this study concerns the extent to which L1 and L2 self-evaluative judgements of French–English bilinguals are impacted comparably. We found that when making a value-laden self-evaluation of their L1 abilities, French-L1 and English-L1 participants homogeneously self-evaluated as maximally proficient—regardless of whether their objective L1 performance, per the French and English LexTALE tasks, was high or low. In other words, when asked to self-evaluate their L1 ability, participants were overall more likely to overestimate their skills, at least according to recorded scores as ascertained by the English LexTALE task. We found this result particularly interesting, as we believe it is likely caused by the restricted range of potential responses that an L1 speaker would entertain as possible when self-evaluating how proficient they are in their more practiced language(s).

When asked to evaluate one’s more practised L1, the automatic self-evaluative response would be to rate oneself as maximally proficient as it likely represents the most practised of the known languages. Accordingly, if a bilingual utilizes and practices a skill on a daily basis such as their L1, there is little reason for that person to evaluate their skills as anything less than maximally proficient. Crucially, we believe this point captures both conceptual and methodological concerns for how self-report questionnaires are often used to evaluate highly proficient, balanced, bilinguals, particularly since the same scales and scale-points are often used to evaluate both L1 and L2 people alike. Indeed, like in many other studies, in this study we asked participants to self-evaluate their language skills using a 7-point Likert scale. Another possibility is that the LexTALE task, while in no doubt accurate for establishing a proxy measure of proficiency that is reliable for L2 speakers, may not be as sensitive when evaluating the more practised L1. Indeed, the LexTALE was originally calibrated to evaluate participants’ L2 proficiency. We believe our results here echo commentaries made by recent work on the need to improve the sensitivity of both subjective and objective tools that are often utilized by language researchers to dichotomize and measure people’s degree of language experience, be it as a categorical construct, a continuous experience, or a combination of both (de Bruin et al., 2017; Gullifer & Titone, 2020; Surrain & Luk, 2019).

Finally, we investigated whether the above-mentioned differences varied when English-L1 and French-L1 participants made usage-based evaluations of their L1. Here, we found a strong correlation between participants’ objective L1 performance and their usage-based L1 evaluations (see Fig. 3, Panel D). However, we found no evidence for an effect of L1 solidarity attitudes on usage-based self-evaluative judgements, indicating that the impact of one’s language solidarity towards the L1, was absent when evaluating a more practised L1. Crucially, this finding aligns with previous literature aiming to demonstrate the advantages of considering language usage as a more reliable proxy for overall language proficiency (Edele et al., 2015; Gullifer & Titone, 2020).

Collectively, the three key results reported here speak to and have implications for the field of bilingualism. They are also relevant to a larger literature on people’s self-evaluation accuracy for any cognitive domain (Ehrlinger, 2008; Kruger, 1999; Larrouy-Maestri et al., 2021; Trofimovich et al., 2016). Over the past two decades, various theoretical explanations like the *Dunning-Kruger* and the *better/worse-than-average* effects have addressed people’s self-assessment inaccuracy in an attempt to address the question of how good people are at assessing themselves. (Oeberst & Imhoff, 2023; Hahn & Harris, 2014). To date, a soft consensus of this work is that people can be somewhat inconsistent when prompted to estimate their abilities across many aspects of human behaviour, from mathematical skills, to the likelihood of developing health problems, to language accentedness and oral comprehensibility (e.g. Ehrlinger et al., 2008; Hulstijn, 2012; Kim et al., 2017; Kruger, 1999; Pronin et al., 2004; Simons, 2013; Trofimovich et al., 2016).

A common finding is that people routinely overestimate or underestimate their abilities relative to others and that these inconsistencies in their estimations tend to vary depending on the ability or skill under evaluation. According to Moore and Small (2007), domains in which people tend to rate themselves *better-than-average* (BTA) are the skill domains in which people generally feel capable and for which they naturally have more practice (e.g. an L1). In contrast, *worse-than-average* (WTA) effects are more likely to be observed or occur for domains in which the skill under evaluation is of a more complex and ambiguous nature (e.g. an L2). However, until recently, not much attention had been paid to how people interpret the term “average” and the possibility that people may have better information about themselves and their ingroups than about other people and other groups (Ehrlinger, 2008; Moore & Small, 2007).

Consistent with this work, we find here that value-laden self-evaluations of language proficiency and usage-based self-evaluations of overall language usage follow a similar pattern to the one found for other abilities in previous studies. We believe that worse-than-average effects may appear more frequently when people self-evaluate their often less practised L2. This might occur because, on average, L2 speakers evaluating their non-native language, by virtue of it being their L2, are generally “less skilled” than L1 speakers. Thus, their evaluation is made by comparing themselves to “the more skilled or native L1 speakers”. This would then be exacerbated in cases where the evaluated language is not only people’s L2, but also the predominant language of the context in which they live. In contrast, in the evaluation of an L1, a better-than-average effect would be expected.

While the results presented paint a coherent picture, we acknowledge several limitations. The first is the correlational nature of the analyses. We believe further experimental and empirical research on the self-evaluative judgement of language experience is needed to shed light on the long-standing question of who people (bilingual or not) compare themselves to when asked to self-assess and report their self-perceived language abilities.

Additionally, in line with prior work investigating the validity of experimental measures to assess language ability, there could be something specific about vocabulary-based behavioural tasks such as LexTALE that make the usage-based assessments more relevant than other types of modality-specific language tasks. In prior studies, calls have been made for a more comprehensive and detailed measurement of self-rated language proficiency measures. Indeed, objective measures of language skills are beneficial as they capture specific components of a person’s receptive and expressive language abilities (e.g. vocabulary knowledge, semantic fluency). However, due to their specificity, objective language proficiency tasks can only capture some components of the language proficiency construct. This is an avenue of research that we are currently pursuing by analysing large-scale multi-site data from a large heterogeneous sample of bilingual participants. We encourage future work to test the generalizability of the results presented here, particularly any conclusions drawn from group-wise comparisons, by analysing a wider range of objective language performance measures and how each of these potential measures patterns with distinct types of self-evaluations for both the L2 and L1. Indeed, in the present study, we primarily relied on questionnaire-based and experimental LexTALE data.

Finally, it is an open question whether the results generalize to other bilingual samples beyond Montréal, Canada, where the contextual salience of English, French, and language attitudes intersect with politics and many other aspects of people’s identity.

In closing, this study suggests that people’s language-specific attitudes can significantly modulate the expected high positive correlation between participants’ value-laden L2 self-evaluations (i.e. *how L2 proficient are you?*) and their objective L2 performance in the LexTALE task. Crucially, this pattern was not observed for usage-based L2 self-evaluations (i.e. *how often do you use your L2?)* for the same people, suggesting that usage-based measures may be less biased by variable inner comparison processes and attitudinal stances towards the skill under evaluation. This has practical implications for the optimal selection and interpretation of self-report measures within bilingualism research. It specifically suggests that usage-based self-evaluations may be more resistant to subjective bias than value-laden self-evaluations. It also suggests that difficulty in accurately self-evaluating one’s language abilities can partially arise from our unique frame-of-reference, which can be impacted by a host of contextual, attitudinal, and individual differences (Titone & Tiv, 2023). Crucially, such phenomena may extend well beyond self-evaluations of language (e.g. Ehrlinger et al., 2008; Fleming, 2024; Hulstijn, 2012; Kruger, 1999; Pronin et al., 2004; Simons, 2013).

## Supplementary Information

Additional file 1.

## Data Availability

The dataset(s) supporting the conclusions of this article is(are) available in the Open Science Framework repository, at https://osf.io/bzra8/.

## References

[CR1] Alicke, M. D., Dunning, D. A., & Krueger, J. (2005). *The Self in Social Judgment*. Psychology Press.

[CR2] Almaatouq, A., Griffiths, T. L., Suchow, J. W., Whiting, M. E., Evans, J., & Watts, D. J. (2022). Beyond playing 20 questions with nature: integrative experiment design in the social and behavioral sciences. *The Behavioral and Brain Sciences*. 10.1017/S0140525X22002874 36539303 10.1017/S0140525X22002874

[CR3] Anderson, J. A. E., Mak, L., Keyvani Chahi, A., & Bialystok, E. (2018). The language and social background questionnaire: assessing degree of bilingualism in a diverse population. *Behavior Research Methods,**50*(1), 250–263. 10.3758/s13428-017-0867-9 28281208 10.3758/s13428-017-0867-9 PMC5591752

[CR4] Anderson, J. A. E., Hawrylewicz, K., & Bialystok, E. (2020). Who is bilingual? Snapshots across the lifespan. *Bilingualism: Language Cognition,**23*(5), 929–937. 10.1017/S1366728918000950

[CR5] Birdsong, D., Gertken, L. M., & Amengual, M. (2012, January 20). *Bilingual Language Profile | An Easy-to-Use Instrument to Assess Bilingualism*. https://sites.la.utexas.edu/bilingual/

[CR6] Bonvin, A., Brugger, L., & Berthele, R. (2023). Lexical measures as a proxy for bilingual language dominance? *Int Rev Appl Linguistics Language Teach,**61*(2), 257–285. 10.1515/iral-2020-0093

[CR7] Bosch, J., & Wilbert, J. (2023). The impact of social comparison processes on self-evaluation of performance, self-concept, and task interest. *Front Education*. 10.3389/feduc.2023.1033488

[CR10] Brysbaert, M. (2013). LexTALE_FR: a fast, free, and efficient test to measure language proficiency in French. *Psychol Belgica,**53*(1), 23–37. 10.5334/pb-53-1-23

[CR11] Cat, C., Kašćelan, D., Prévost, P., Serratrice, L., Tuller, L., & Unsworth, S. (2023). Q-BEx consortium how to quantify bilingual experience? findings from a delphi consensus survey. *Bilingual Lang Cognit,**26*(1), 112–124.

[CR9] de Bot, K. (2019). Defining and Assessing Multilingualism. In J. W. Schwieter & M. Paradis (Eds.), *The Handbook of the Neuroscience of Multilingualism* (1st ed., pp. 1–18). Wiley. 10.1002/9781119387725.ch1

[CR12] de Bruin, A., Carreiras, M., & Duñabeitia, J. A. (2017). The BEST dataset of language proficiency. *Frontiers in Psychology*. 10.3389/fpsyg.2017.00522 28428768 10.3389/fpsyg.2017.00522 PMC5382213

[CR13] Dragojevic, M., Fasoli, F., Cramer, J., & Rakić, T. (2021). Toward a century of language attitudes research: looking back and moving forward. *J Lang Social Psychol,**40*(1), 60–79. 10.1177/0261927X20966714

[CR14] Ehrlinger, J. (2008). Skill level, self-views and self-theories as sources of error in self-assessment: sources of error in self-assessment. *Soc Personal Psychol Compass,**2*(1), 382–398. 10.1111/j.1751-9004.2007.00047.x

[CR15] Feng RY, Tiv M, Kutlu E, Gullifer JW, Palma P, O’Regan E, Vingron N, Doucerain MM, Titone D. A systems approach to multilingual language attitudes: A case study of Montréal, Québec, Canada. Int J Bilingual. 2024;28(3):454-78 10.1177/13670069221133305 PMC11178480 38881568

[CR16] Ferré, P., & Brysbaert, M. (2017). Can Lextale-ESP discriminate between groups of highly proficient Catalan-Spanish bilinguals with different language dominances? *Behavior Research Methods,**49*(2), 717–723. 10.3758/s13428-016-0728-y 27004486 10.3758/s13428-016-0728-y

[CR17] Flake, J. K., Davidson, I. J., Wong, O., & Pek, J. (2022). Construct validity and the validity of replication studies: A systematic review. *American Psychologist,**77*(4), 576–588. 10.1037/amp0001006 35482669 10.1037/amp0001006

[CR18] Fleming, S. M. (2024). Metacognition and confidence: a review and synthesis. *Ann Rev Psychol,**75*(1), 241–268. 10.1146/annurev-psych-022423-032425 37722748 10.1146/annurev-psych-022423-032425

[CR19] Garcia, D. L., & Gollan, T. H. (2022). The MINT Sprint: exploring a fast administration procedure with an expanded multilingual naming test. *Journal of the International Neuropsychological Society,**28*(8), 845–861. 10.1017/S1355617721001004 34463235 10.1017/S1355617721001004 PMC8882711

[CR20] Gardner, R. C., & Lambert, W. E. (1972). Attitudes and motivation in second language learning (Vol. 786). Rowley Newbury.

[CR21] Genesee, F., & Holobow, N. E. (1989). Change and stability in intergroup perceptions. *J Lang Social Psychol,**8*(1), 17–38. 10.1177/0261927X8900800102

[CR500] Giles, H., Taylor, D. M., & Bourhis, R. (1973). Towards a theory of interpersonal accommodation through language: Some Canadian data. *Language in Society, 2*(2), 177–192

[CR22] Giles, H., & Coupland, N. (1991). Language: Contexts and consequences. Open University Press.

[CR23] Giles, H., & Ryan, E. B. (1982). Prolegomena for developing a social psychological theory of language attitudes. In E. Ryan & H Giles. (Eds.), Attitudes towards language variation (pp. 208–223). Edward Arnold.

[CR24] Gollan, T. H., Weissberger, G. H., Runnqvist, E., Montoya, R. I., & Cera, C. M. (2012). Self-ratings of spoken language dominance: A Multilingual Naming Test (MINT) and preliminary norms for young and aging Spanish-English bilinguals. *Bilingual Lang Cognit,**15*(3), 594–615. 10.1017/S1366728911000332 10.1017/S1366728911000332 PMC4212892 25364296

[CR25] Gullifer, J. W., & Titone, D. (2020). Characterizing the social diversity of bilingualism using language entropy. *Bilingual: Lang Cognit,**23*(2), 283–294. 10.1017/S1366728919000026

[CR26] Gullifer, J. W., Kousaie, S., Gilbert, A. C., Grant, A., Giroud, N., Coulter, K., Klein, D., Baum, S., Phillips, N., & Titone, D. (2021). Bilingual language experience as a multidimensional spectrum: associations with objective and subjective language proficiency. *Appl Psycholinguistics,**42*(2), 245–278. 10.1017/S0142716420000521

[CR27] Hahn, U., & Harris, A. J. L. (2014). Chapter Two - What Does It Mean to be Biased: Motivated Reasoning and Rationality. In B. H. Ross (Ed.), *Psychology of Learning and Motivation* (Vol. 61, pp. 41–102). Academic Press. 10.1016/B978-0-12-800283-4.00002-2

[CR28] Heilenman, L. (1990). Self-assessment of second language ability: the role of response effects. *Language Testing,**7*(2), 174–201. 10.1177/026553229000700204

[CR29] Hulstijn, J. H. (2012). The construct of language proficiency in the study of bilingualism from a cognitive perspective. *Bilingual: Lang Cognit,**15*(2), 422–433. 10.1017/S1366728911000678

[CR30] Izura, C., Cuetos, F., & Brysbaert, M. (2014). Lextale-Esp: A test to rapidly and efficiently assess the Spanish vocabulary size. *Psicologica,**35*(1), 49–66.

[CR31] Kałamała, P., Senderecka, M., & Wodniecka, Z. (2022). On the multidimensionality of bilingualism and the unique role of language use. *Bilingual: Lang Cognition,**25*(3), 471–483. 10.1017/S1366728921001073

[CR32] Kašćelan, D., Prévost, P., Serratrice, L., Tuller, L., Unsworth, S., & Cat, C. D. (2022). A review of questionnaires quantifying bilingual experience in children: Do they document the same constructs? *Bilingualism Lang Cognition,**25*(1), 29–41. 10.1017/S1366728921000390

[CR33] Kaushanskaya, M., Blumenfeld, H. K., & Marian, V. (2020). The language experience and proficiency questionnaire (LEAP-Q): Ten years later. *Bilingual Lang Cognition,**23*(5), 945–950. 10.1017/S1366728919000038 10.1017/s1366728919000038 PMC7899192 33628083

[CR34] Kim, Y.-H., Kwon, H., & Chiu, C.-Y. (2017). The better-than-average effect is observed because “average” is often construed as below-median ability. *Frontiers in Psychology,**8*, 898. 10.3389/fpsyg.2017.00898 28690555 10.3389/fpsyg.2017.00898 PMC5479883

[CR35] Kircher, R. (2014). Thirty years after Bill 101: A contemporary perspective on attitudes towards English and French in Montreal. *Canadian J Appl Linguistics,**17*(1), 20–50.

[CR36] Kircher, R., Quirk, E., Brouillard, M., Ahooja, A., Ballinger, S., Polka, L., & Byers-Heinlein, K. (2022). Quebec-based parents’ attitudes towards childhood multilingualism: evaluative dimensions and potential predictors. *J Lang Social Psychol,**41*(5), 527–552. 10.1177/0261927X221078853 10.1177/0261927X221078853 PMC9421620 36051630

[CR46] Kircher, R., & Zipp, L. (Eds.). (2022). Research Methods in Language Attitudes. In *Research Methods in Language Attitudes* (pp. 1–2). Cambridge University Press. 10.1017/9781108867788

[CR37] Kruger, J. (1999). Lake Wobegon be gone! The “below-average effect” and the egocentric nature of comparative ability judgments. *J Personal Social Psychol,**77*(2), 221–232. 10.1037/0022-3514.77.2.221 10.1037//0022-3514.77.2.221 10474208

[CR38] Larrouy-Maestri, P., Wang, X., Vairo Nunes, R., & Poeppel, D. (2021). Are You Your Own Best Judge? On the Self-Evaluation of Singing. *J Voice*. 10.1016/j.jvoice.2021.03.028 10.1016/j.jvoice.2021.03.028 34011458

[CR39] Lemhöfer, K., & Broersma, M. (2012). Introducing LexTALE: a quick and valid lexical test for advanced learners of english. *Behavior Research Methods,**44*(2), 325–343. 10.3758/s13428-011-0146-0 21898159 10.3758/s13428-011-0146-0 PMC3356522

[CR40] Li, P., Zhang, F., Yu, A., & Zhao, X. (2020). Language history questionnaire (LHQ3): an enhanced tool for assessing multilingual experience. *Bilingual: Lang Cognition,**23*(5), 938–944. 10.1017/S1366728918001153

[CR41] MacIntyre, P. D., Noels, K. A., & Clément, R. (1997). Biases in self-ratings of second language proficiency: the role of language anxiety. *Language Learn,**47*(2), 265–287. 10.1111/0023-8333.81997008

[CR42] Marian, V., Blumenfeld, H. K., & Kaushanskaya, M. (2007). The language experience and proficiency questionnaire (LEAP-Q): assessing language profiles in bilinguals and multilinguals. *Journal of Speech, Language, and Hearing Research,**50*(4), 940–967. 10.1044/1092-4388(2007/067) 17675598 10.1044/1092-4388(2007/067)

[CR43] Mathôt, S., Schreij, D., & Theeuwes, J. (2012). OpenSesame: an open-source, graphical experiment builder for the social sciences. *Behavior Research Methods,**44*(2), 314–324. 10.3758/s13428-011-0168-7 22083660 10.3758/s13428-011-0168-7 PMC3356517

[CR44] Moore, D. A., & Small, D. A. (2007). Error and bias in comparative judgment: On being both better and worse than we think we are. *J Person Soc Psychol,**92*(6), 972–989. 10.1037/0022-3514.92.6.972 10.1037/0022-3514.92.6.972 17547483

[CR45] Pronin, E., Gilovich, T., & Ross, L. (2004). Objectivity in the eye of the beholder: divergent perceptions of bias in self versus others. *Psychological Review,**111*(3), 781–799. 10.1037/0033-295X.111.3.781 15250784 10.1037/0033-295X.111.3.781

[CR47] Rothman, J., Bayram, F., DeLuca, V., Pisa, G. D., Duñabeitia, J. A., Gharibi, K., Hao, J., Kolb, N., Kubota, M., Kupisch, T., Laméris, T., Luque, A., van Osch, B., Soares, S. M. P., Prystauka, Y., Tat, D., Tomić, A., Voits, T., & Wulff, S. (2023). Monolingual comparative normativity in bilingualism research is out of “control”: arguments and alternatives. *Appl Psycholinguistics,**44*(3), 316–329. 10.1017/S0142716422000315

[CR48] Ryan, E. B., Giles, H., & Sebastian, R. J. (1982). An integrative perspective for the study of attitudes toward language variation. In E. B. Ryan & H. Giles (Eds.), *Attitudes towards language variation: Social and applied contexts* (pp. 1–19). Edward Arnold.

[CR49] Siegelman, N., Elgort, I., Brysbaert, M., Agrawal, N., Amenta, S., Arsenijević Mijalković, J., Chang, C. S., Chernova, D., Chetail, F., Clarke, A. J. B., Content, A., Crepaldi, D., Davaabold, N., Delgersuren, S., Deutsch, A., Dibrova, V., Drieghe, D., Filipović Đurđević, D., Finch, B., & Kuperman, V. (2023). Rethinking First Language-Second Language Similarities and Differences in English Proficiency: Insights From the ENglish Reading Online (ENRO) Project. *Language Learning*. 10.1111/lang.12586

[CR50] Simons, D. J. (2013). Unskilled and optimistic: Overconfident predictions despite calibrated knowledge of relative skill. *Psychonomic Bulletin & Review,**20*(3), 601–607. 10.3758/s13423-013-0379-2 23345139 10.3758/s13423-013-0379-2

[CR51] Surrain, S., & Luk, G. (2019). Describing bilinguals: a systematic review of labels and descriptions used in the literature between 2005–2015. *Bilingual: Lang Cognition,**22*(2), 401–415. 10.1017/S1366728917000682

[CR52] Tajfel, H., & Turner, J. C. (1986). The social identity theory of intergroup behavior. In S. Worchel & W. G. Austin (Eds.), *Psychology of intergroup relations* (pp. 7–24). Nelson-Hall.

[CR53] Titone, D., & Tiv, M. (2023). Rethinking multilingual experience through a systems framework of Bilingualism. *Bilingual Lang Cognition,**26*(1), 1–16. 10.1017/S1366728921001127

[CR54] Titone, D., Hernández-Rivera, E., Iniesta, A., Beatty-Martínez, A. L., & Gullifer, J. W. (2024). Are language–cognition interactions bigger than a breadbox? Integrative modeling and design space thinking temper simplistic questions about causally dense phenomena. *The Behavioral and Brain Sciences,**47*, e60. 10.1017/S0140525X23002145 38311462 10.1017/S0140525X23002145

[CR55] Tiv, M., Kutlu, E., Gullifer, J. W., Feng, R. Y., Doucerain, M. M., & Titone, D. A. (2022). Bridging interpersonal and ecological dynamics of cognition through a systems framework of bilingualism. *J Exp Psychol: General,**151*(9), 2128–2143. 10.1037/xge0001174 10.1037/xge0001174 35113642

[CR56] Tomoschuk, B., Ferreira, V. S., & Gollan, T. H. (2019). When a seven is not a seven: self-ratings of bilingual language proficiency differ between and within language populations. *Bilingual: Lang Cognit,**22*(3), 516–536. 10.1017/S1366728918000421

[CR57] Trofimovich, P., Isaacs, T., Kennedy, S., Saito, K., & Crowther, D. (2016). Flawed self-assessment: investigating self- and other-perception of second language speech. *Bilingual Lang Cognition,**19*(1), 122–140. 10.1017/S1366728914000832

[CR58] Veríssimo, J. (2021). Analysis of rating scales: a pervasive problem in bilingualism research and a solution with Bayesian ordinal models. *Bilingual Lang Cognition,**24*(5), 842–848. 10.1017/S1366728921000316

[CR61] Wagner, D., Bialystok, E., & Grundy, J. G. (2022). What is a language? Who Is Bilingual? Perceptions Underlying self-assessment in studies of Bilingualism. *Frontiers in Psychology,**13*, 863991. 10.3389/fpsyg.2022.863991 35645938 10.3389/fpsyg.2022.863991 PMC9134110

[CR62] Wigdorowitz, M., Pérez, A. I., & Tsimpli, I. M. (2022). Sociolinguistic context matters: exploring differences in contextual linguistic diversity in South Africa and England. *Int Multilingual Res J,**16*(4), 345–364. 10.1080/19313152.2022.2069416

[CR63] Wigdorowitz, M., Pérez, A. I., & Tsimpli, I. M. (2023). A holistic measure of contextual and individual linguistic diversity. *Int J Multilingual*, 1–19. 10.1080/14790718.2020.1835921

