# Supplementary material for: Self-evaluations and the language of the beholder: objective performance and language solidarity predict L2 and L1 self-evaluations in bilingual adults
Source: Cogn Res Princ Implic. 2024 Nov 4;9:75. doi: 10.1186/s41235-024-00592-4 (PMC11535130; doi:10.1186/s41235-024-00592-4)
Supplement: Supplementary file 1 — Additional file 1. [file 41235_2024_592_MOESM1_ESM.docx]

**Supplemental Materials: Self-Evaluations and the Language of the Beholder: Objective Performance and Language Solidarity Predict L2 and L1 Self-Evaluations in Bilingual Adults**

**Table S1. Complete set of questions used to index L1 and L2 attitudinal language solidarity.**

| **Question** |
| --- |
| Knowing *Lx* is an important part of my personal identity. |
| Speaking *Lx* gives me a sense of community. |
| I like who I am when I speak *Lx*. |
| Speaking in *Lx* increases the value and prestige of what I say. |
| *Lx* makes me feel secure. |
| I feel true to myself when I speak *Lx*. |
| In a new social context, I prefer to be addressed in *Lx*... |
| I feel motivated to speak *Lx*... |

*Note.* Lx is used here as a placeholder. In the questionnaire, participants saw either English or French in place of Lx.

**Table S2. Participants self-reported known language combinations.**

| **Language Combination** | **Count** |
| --- | --- |
| English and French | 23 |
| English and French (+ Spanish) | 13 |
| English and French (+ Italian) | 4 |
| English and French (+ Italian and Spanish) | 3 |
| English and French (+ German) | 3 |
| English and French (+ Mandarin and Spanish) | 3 |
| English and French (+ Creole) | 3 |
| English and French (+ Japanese and Spanish) | 2 |
| English and French (+ Arabic) | 2 |
| English and French (+ Other languages) | 7 |

***Table S3. Participants self-reported known languages.***

| **Language** | **Count** |
| --- | --- |
| English | 62 |
| French | 62 |
| Spanish | 28 |
| Italian | 7 |
| German | 3 |
| Arabic | 3 |
| Dutch | 1 |
| Mandarin | 2 |
| Hebrew | 2 |
| Korean | 1 |
| Khmer | 1 |
| Japanese | 1 |

*Note.* The counts reported here represent each of the self-reported known languages identified in the full dataset. Since a single participant was allowed to report knowledge of up to 4 distinct languages, the counts identified here are greater than the total number of participants in the final sample. Thus, in this table, a single participant was allowed to be counted up to four times.

**Table S4. Participants place-of-birth (country)**

| **Place of Birth** | **Count** |
| --- | --- |
| Canada (*Quebec*) | 34 |
| Canada (*Outside of Quebec*) | 10 |
| United States of America | 4 |
| Morocco | 2 |
| Haiti | 1 |
| UK | 2 |
| France | 8 |
| Cambodia | 1 |
| Belgium | 1 |
| Algeria | 1 |
